# Generation Alpha’s expectations at work: Well-being, pay and sense of agency

**DOI:** 10.1371/journal.pone.0336725

**Published:** 2026-04-30

**Authors:** Marzena Syper-Jędrzejak, Katarzyna Wojtaszczyk, Patrycja Mizera, Izabela Różańska-Bińczyk, Paweł Łuczak

**Affiliations:** Department of Human Resources Management, Faculty of Management, University of Lodz, Lodz, Poland; Grigore T Popa University of Medicine and Pharmacy Iasi: Universitatea de Medicina si Farmacie Grigore T Popa lasi, ROMANIA

## Abstract

In contemporary organizations, representatives of different generations meet. Many publications have been written about the work style, expectations, and needs of representatives of previous generations, but we notice a certain gap concerning the youngest participants of the labor market, i.e., Generation Alpha. The aim of the publication was to find answers to the questions about the requirements for remuneration and well-being at work of young people in Poland, whether these two groups of expectations are causally related, and what is the role of the sense of agency of representatives of Generation Alpha in this causal-effect system. We surveyed 446 respondents aged 19–22, selected on a quota basis. The results of the study confirmed that the expectations of the youngest players in the labor market go in many directions: first, toward earning “good” money, secondly – well-being at work is important. We confirmed that the expectation of young people regarding satisfactory remuneration is related to the expectations (and implicitly also the search for) of an employer who will ensure well-being at work. However, it was not possible to directly confirm the mediating role of the high sense of agency in the relationship between expectations about pay and well-being at work.

## Introduction

Employers are currently dealing with highly diversified age groups, with different attitudes to work, different understandings of their own development, and loyalty to the company. Each age group brings with it a different baggage of experiences and aspirations related to professional life, which is expressed in different value systems, approaches to work, and a hierarchy of needs [1–3]. An accurate understanding of employee needs and motivations is a condition for developing effective management strategies, and each generation requires a different approach [4,5] if the organization wants to fully use the potential of their knowledge and skills.

A particular challenge for employers and managers is the youngest generation, which is starting its professional activity, Generation Alpha, raised in the world of modern technologies (smartphones, tablets, and social media channels), growing up in relatively comfortable economic and social conditions, and even in a “culture of excess” [6–8]. This generation has so far been considered rather in terms of recipients of educational services and young consumers, especially in the field of modern technologies [7,9] meanwhile, the oldest Alphas are already active on the labor market. This means that there is a research gap (mainly knowledge and theoretical gap) regarding the clear connection between expectations regarding remuneration and well-being in the workplace of representatives of Generation Alpha.

To fill the observed research gap, we undertook empirical research. Inspired by the predictions made by other researchers about how young people will behave as employees and by reports from the global [10–12] or local labor market [13] we tried to understand the expectations of this generation regarding the workplace. These expectations, similarly to other generations, do not end with remuneration. Our attention was drawn to the interest of young people in the broadly understood comfort of life, place of study or work, the expectation that the environment will flexibly adapt to them and, by offering “personalized” solutions, meet their needs, in other words, provide them with well-being [8,12,14,15]. Following the findings of dos Reis [16] and Apaydin and Kaya [17] we assume that autonomy in decision-making and action, as well as a kind of “self-determination”, are also important for the young generation. In this context, we considered the sense of agency to be an important factor that determines life choices and actions and significantly contributes to the achievement well-being [18,19]. Although the issues of the need for autonomy of action or ensuring comfort and well-being in young people appear in the findings of the researchers cited earlier, we did not find studies explaining the relationship between young people’s sense of agency and their expectations regarding the workplace in terms of remuneration or ensuring well-being.

The main goal of the presented study was to find out what expectations young people have regarding remuneration and well-being at work, whether these two groups of expectations are causally related, and to check the role of Generation Alpha’s sense of agency in this causal system. We built a model including these variables (Generation Alpha’s expectations regarding remuneration and ensuring well-being at work, with a sense of agency as a moderator) and checked whether it meets the requirements of reliability and validity.

## Literature review and hypotheses development

### Generation Alpha enters the job market

Each generation seems to be skeptical of younger generations; Socrates and Plato were already critical of the youth of their time [20]. Until about the middle of the 20th century, works on young people were usually written from the perspective of older generations. It was not until the publication *of Generation X* by Hamblet and Deverson [21] that a trend began to characterize generations by giving a voice to their representatives [22–24]. Currently, the voices of young people themselves are published by consulting firms in numerous reports on the youngest generations [11,25,26].

Human resources today in organizations include five distinct generational groups (Baby Boomer, X, Y, Z, Alpha), with distinctly different attitudes, motivations, and priorities [27–29]. In this discourse, a generation is understood as a group (cohort) sharing a certain point in history, a collective personality with a similar life, and values shaped by historical events or life circumstances [30]. When discussing generational groups, it should be noted that generation classifications are ambiguous [31] with generational cohorts usually covering a period of 15–20 years [32]. The basis for distinguishing generations is significant events or cultural experiences that shape generational affiliation, the so-called generational signposts [33] that leave a lasting effect, whether social, economic, or political. Of course, it should be noted that these important historical events that can define a generational cohort vary significantly depending on location or experience [34] – for example, the perception of the 2001 WTC attack was different for an American teenager at the time and different for his peer in Japan. Incidentally, we also have many positions questioning the validity of empirical evidence for the existence of actual generational differences [35–37]. The British Council report [38] draws attention, for example, to the large diversity of the generation of young adults due to regional differences, education and level of wealth – which is worth remembering when talking about the generation as a whole. This also applies to Generation Alpha, a term introduced by McCrindle [39]. In his opinion, Generation Alpha (in developed countries) is made up of people born between 2010 and 2024 [39] Ryan [19] defines the time frame of this generation in a similar way. However, there are reasons to adopt a different time perspective. For example, it is pointed out that the pace of technological change is so fast that it leads to an increasingly narrow definition of generational cohorts. The rigid 20-year timeframe adopted for generations becomes methodologically problematic [40] If we use the criterion of technological conditions in which it grew up to determine a generation, generations will cover shorter and shorter time intervals. In addition, the literature analysis shows that people born after 2000 already show traits attributed to the Alpha generation, such as: high digital proficiency, individualism, emotionality and cognitive dynamism, which distinguishes them from earlier cohorts [ 8,41]. Even McCrindle, who coined the term “Generation Alpha,” admits that cut-off dates are fluid and context-dependent [42]. This opens up space for us to adopt an alternative approach, in which the year 2000 could be the beginning of a new cohort (Gen Alpha), especially if we take into account the impact of digitalization, globalization and lifestyle change.

The representatives of Generation Alpha (which mainly concerns the youth of the so-called developed countries) are learning and gaining their first professional experience in the world of Industry 4.0 and the digital transformation of enterprises, accelerated by the COVID-19 pandemic, which is fundamentally changing the nature of work, the way of doing business and the very functioning of societies [43–45]. These factors influence the labor market, along with social changes, shaping the preferences of young people, low interest in working in “real” sectors of the economy (production, agriculture, etc.) or a preference for remote work [15]. In addition, there is the use of technology in everyday entertainment, education, or work [6–9]. The findings to date show that young people are enterprising [46], creative [16,47], who require flexible organizational solutions from employers that meet their needs [14]. Local Polish studies indicate that young people also expect job security and fair treatment, as well as care for providing them with comfortable working conditions [13,48]. They also value authenticity (understood as honest and free expression of oneself, one’s views and opinions) and autonomy [16,17].

When talking about the expectations of young people regarding work (current or future), it is impossible to ignore the issue of remuneration. For Polish representatives of Generation Alpha, just like their older colleagues, the Zetas before them, the role of high remuneration is crucial, which is at the top of the rankings of expectations towards the employer [48,49].

On the other hand, we have a large group of young people who do not want to work or study [50,51]. Healthcare professionals report a large scale of mental health problems: the number of adolescents using psychiatric care is growing, and national systems for the prevention of mental health of young people are overloaded [52,53]. For example, the number of young patients suffering from ecological anxiety and habitually worrying about the environment is growing [54,55]. The COVID-19 pandemic has changed people’s functioning in many areas of life, and we are dealing with common mental disorders (CMD) [56]. Exposure to cyberbullying is also a particular threat to young people [57].

Identifying the expectations and needs of young people (as well as the concerns and problems that preoccupy them) can help employers who want to hire Generation Alpha to design a job offer that will be attractive to young employees.

An important asset of the employer for young people is to create working conditions in which the employee can feel comfortable, both physically, mentally and socially – and the factors that build this comfort are different across generations [58,59]. For the youngest, for example, care for mental health and internal well-being (i.e., related to emotional balance) in the workplace will be important because, as mentioned earlier, they are informationally overloaded and particularly susceptible to cyber violence, ecological anxiety and other “civilizational” threats. It is estimated that well-being at work will become a key issue and a need for this generation [60].

### Well-being in the workplace and sense of agency

Organizations invest in health and well-being initiatives to positively impact health, and productivity, or reduce employee turnover [61]. Although the terms wellness and well-being are popular in the scientific debate, they are interpreted differently and do not have a clear definition at the conceptual level [62–65].

The term well-being appeared in the definition of health formulated in 1948 by the World Health Organization. This definition described health as a state of complete physical, mental, and social well-being and not merely the absence of disease or infirmity [66,67]. Attempts to define well-being were based on an attempt to identify and describe the ‘components’ of well-being. For example Zheng et al. [65] noted that well-being can be said to occur when the relations between a person’s resources and challenges are in relative balance.

A related term to well-being is wellness, first used in the context of lifestyle in the 1950s by Dunn and described by him as a way of life that aims to achieve well-being through the harmony of body, mind, and spirit [68].

In the literature on the subject, well-being is understood in two broad perspectives, objective and subjective. The first includes traditional neoclassical measures of the “good life”, such as: life expectancy, low crime risk, low poverty rates and good quality of the natural environment [69]. This is consistent with the proposal of the construct of objective components of life satisfaction, including, for example, income, professional status, or availability of resources in the human environment. It should be noted that objective well-being can be understood as the quality of life [70] but it can also be interpreted completely differently, e.g., in the hedonistic theory of objective happiness according to Kahneman [71,72]. It also seems that determining objective measures of well-being may be difficult because most tools for describing or measuring well-being are based on subjective self-report [73].

The second perspective on well-being, which is of particular interest to us due to the set goals, concerns subjective indicators of a given person’s well-being, i.e., attempts to assess what people think about their lives, relationships, or work. This is consistent with the three-dimensional definition of health proposed by the WHO, in which health consists of physical, mental, and social factors. Importantly, well-being (or: well-being) does not only include a description of the previously mentioned individual aspects but is an integrated whole, enabling an individual to cope with life circumstances and achieve an optimal state of existence [74,75]. In this paradigm of thinking about well-being, the emphasis is on lifestyle behaviors that go beyond basic (physical) health and lead to optimal states in physical, mental, and social dimensions [76,77].

Despite these attempts to develop measures to determine the level of well-being, a systematic review [78] revealed that there is no uniform definition of well-being in the subjective sense, and descriptions of well-being vary widely. For example, according to Diener’s team [79], well-being is a cognitive and affective assessment of life, which may include emotional reactions to events, as well as a cognitive assessment of one’s own satisfaction with life and sense of fulfillment. Subjective well-being consists of affective components (experiencing positive emotions, low frequency of experiencing negative emotions) and cognitive components (high satisfaction with life). Keyes and Waterman [80] understood well-being as a multidimensional phenomenon, encompassing at least three aspects: (1) psychological well-being, (2) emotional well-being, and (3) social well-being. Well-being is, therefore, more than positive emotional states; it is also positive functioning in the psychological and social sphere. In this approach, subjective well-being corresponds to mental health [81] Seligmann [82] proposes a model in which well-being is an operationalization of human flourishing and distinguishes five dimensions: 1) positive emotion, 2) achievement, 3) meaning and purpose, 4) relationships with others, and 5) engagement. Seligman emphasizes that each of these elements can be measured independently of the others, each contributes to well-being, and striving for one dimension is not dictated by the desire to achieve another [83].

In the understanding of well-being, emphasis is therefore placed on the multidimensional nature of well-being. Looking for common areas for well-being models functioning in the literature, it can be noticed that this construct assumes the following levels of “good” human functioning, within the limits defined by the individual’s capabilities: starting from (1) the level of physical well-being, (2) mental, (3) emotional, (4) social, up to (5) the level of spiritual well-being [83]. Recognizing and practicing these six dimensions of well-being in workplaces, as well as in educational institutions by employees, students, and athletes, can shape a happier society with a better attitude [84].

However, it is impossible to describe and analyze the areas of subjective well-being without considering the aforementioned external conditions that shape personal behaviors, strategies, and assessments [85]. Such external influences include the professional environment, which is both an important component of the overall assessment of well-being and the space in which this well-being is realized.

In management sciences, the individual psychological well-being of an employee is placed against the background (or in the environment) of the organization, while at the same time transferring the burden of responsibility for well-being from the employee to the employer [86]. The role of management in supporting well-being therefore seems to be providing employees with physical, mental, and social resources in the face of challenges posed by working conditions or the nature of tasks [87]. A separate stream of research on employee well-being has been distinguished in the management literature [88,89]. Employee well-being is defined as the overall experience and functioning of employees in: 1. The physical dimension – with particular emphasis on ergonomics and work comfort, physical safety, and health protection at work; 2. psychological, in the sense of emotional balance, the ability to meet important needs, coping with stress; 3. relational – including satisfaction with cooperation, support, and trust in the workplace [90–92].

The topic of well-being is so important that there is evidence of a link between a high level of employee well-being and other significant variables, such as productivity, engagement or the creation of a desirable organizational culture [93,94]. However, there is are data indicating that a high level of employee well-being does not always translate into their current productivity. In a review by Van de Voorde [89] which analyzed 36 studies on the effects of wellness activities, Dutch researchers concluded that the level of organizational productivity and employee health well-being seem to be “conflicted”, i.e., a high level of one lowers the level of the other indicator. On the other hand, employees who have been subjected to different HR practices report higher levels of well-being [89]. Regarding productivity itself, on the other hand, its reduction is observed in connection with presenteeism or health-risk elements of employees’ lifestyles, such as smoking, drinking alcohol, and obesity, i.e., areas classified as determinants of well-being [95]. These issues are also the subject of interest of organizations, implemented through, for example, creating solutions in the field of health prevention or promoting a healthy lifestyle among employees.

There are certain conditions that are conducive to the development of employee well-being. The basic ones include matching employees in terms of their competencies and personality traits with the tasks assigned to them and the nature of their work. Another, quite obvious, is creating the best possible working conditions and the so-called empowerment of employees, enabling them to set their own goals, make decisions, and solve problems within their scope of responsibility and authority [96,97]. A review of research and literature indicates that factors that build well-being in an organization include the use of organizational methods (e.g., HRM), which increase the employee’s sense of autonomy and are developmental in nature, increase the sense of meaning and significance of work, and build self-acceptance and a sense of achievement. This may mean abandoning competitive methods and comparing employees or limiting the use of corporate social media for this purpose. The key task of managers is to maintain a balance between challenges and physical, mental, and social resources, e.g., by providing them to the employee, ensuring sustainable development, including in the field of new technologies and related challenges. An organization managed in this way optimizes, not maximizes, the level of employee productivity (assuming that organizational culture can play a mediating role between productivity and wellness). Only on such a basis, specific wellness programs dedicated to employees, providing emotional support or support in leading a healthy lifestyle, will bring optimal results, with the appropriate support of management staff and designated experts [98,99]. There is evidence that the sense of well-being of employees, in turn, decreases when an organization introduces a culture of competition into management practices and increased stress appears in connection with inevitable comparisons [100]. Similarly, for example, excessive use of corporate social media, pressure on the employee to be in constant contact [101]. To estimate the real effects of wellness activities and factors that influence the level of well-being, some researchers suggest adding the impact of organizational culture to the analyzes, as an important mediator of wellness [94,102]. Work is satisfying when relationships between coworkers are good, the atmosphere is pleasant, and the employee himself is appreciated and rewarded [103].

Well-being at work depends on both organizational factors, related to the work environment, and individual factors – inherent in the individual. Individual factors are mainly related to personality traits [104–106]. Awareness of how important well-being at work is and how to build it can be of great importance for employees’ professional and life success.

Among the internal determinants of well-being that may explain its formation and maintenance, we can point to the sense of agency [18,107].The sense of agency is the subject of increasing attention from management scientists. It corresponds to the ability to attribute to oneself the initiation and authorship of an action [108], to associate specific consequences with a specific action [109] and is considered a key point in the development of consciousness. Agency is otherwise known as the focus on one’s own self and oneself as the implementer of goals [110,111] or the ability to independently define oneself while searching for answers to questions about the meaning of existence, life plans, needs and aspirations.

The sense of agency is semantically linked to other constructs. The first works on the sense of agency were related to Bandura’s findings on the sense of self-efficacy. Bandura’s sense of self-efficacy [112] is the belief in one’s own ability to organize and carry out the actions necessary to manage future situations and the belief that one is able to take effective actions necessary to produce the desired effect, even when obstacles appear. In other words, it is a deep conviction about one’s own ability to achieve success in a specific situation, the assessment that one is able to mobilize oneself to cope with a given task, challenge, or problem. In this approach, the sense of self-efficacy is not the certainty that our specific action will succeed but concerns the certainty (belief) that one is able to achieve such success at all. Therefore, it is a motive to take action. On the other hand, the lack of such faith in one’s own abilities blocks further activities [9]. Empirical studies have shown that a high sense of self-efficacy is a good predictor of many different activities and behaviors related to long-term and effort-requiring activities such as practicing sports, losing weight, or quitting addiction [113].

If people believe that they have the skills to cope with a situation, they feel strong and confident. In this sense, the sense of agency is close to Allport’s [114] concept of independence, which emphasizes a mature personality and a sense of coherent identity that finds expression in a realistic perception of oneself, the ability to achieve and experience success, and in coping with failure.

The sense of agency, next to self-determination or decision-making, is connected (and by some is identified) with autonomy. The concept of autonomy can be used in various contexts, but from our point of view, the developmental perspective and reference to the developmental period in which representatives of the Alpha generation find themselves are particularly important. The period of adolescence is a transitional period between childhood and adulthood, and from the point of view of developmental tasks, the ability to independently determine the direction of one’s own life and its purpose, as well as achieving emotional independence from parents and other adults, becomes crucial here. In other words, the ability to make autonomous decisions and choices is formed in adolescence [115]. In turn, Erikson analyzes autonomy in the context of a developmental crisis taking place in the process of identity formation [116]. This researcher assumed that the conviction of the subject’s own independence develops in situations indicating that he or she has control in connection with the manifestation of behavior appropriate to the situation. The process of developing autonomy can be observed from birth and is the basis for shaping the image of one’s own „I”, that is, distinguishing and integrating one’s own structure through interactions with the environment. The pursuit of independence of decisions and actions by young people is therefore a natural phenomenon, very characteristic of the period of adolescence. At this time, the individual undertakes actions aimed at integrating previous knowledge about oneself and the world, which constitutes the foundation for the crystallization of one’s identity [117,118]. Manifestations of the pursuit of autonomy visible in the behavior of children include, among others, the ability to express one’s own needs and expectations, the sense of having something of one’s own, the independence in performing activities, the desire to make decisions, demonstrating one’s own opinion or desires, and a sense of agency [119].

The sense of agency is therefore a belief in one’s own efficacy, resulting in effectiveness in action, the ability to achieve chosen goals and a focus on effective performance of undertaken actions. Some people place agency in the range of personal competences [18].

The sense of agency is related to building well-being. In their concept of self-determination, Ryan and Deci noted that the need for autonomy (agency) is crucial for shaping the subjective psychological well-being of a person, which is the basis of a homeostatic and coherent personality and effective action [19]. An autonomous individual will therefore be characterized by the ability to manage their own behavior and make decisions, considering personal needs [19]. Agency affects the ability to self-control, which in turn has a direct impact on mental and physical health, being the foundation for building one’s well-being [120]. In the model of Welzel and Inglehart [121] the sense of agency is a key factor in life satisfaction and well-being, especially in the context of work and social development. Taking into account the broader, organizational context – some studies indicate that creating working conditions in which employees can demonstrate agency results in higher employee well-being [122]. This can also be translated into the area of professional decisions and career management. There is evidence that a high sense of agency increases social and civic activity, and at the same time is reinforced by the actions taken, especially collective ones [119].

It can be assumed that the need to build or regain agency may increase in developed societies, due to the growing uncertainty and unpredictability of more and more dimensions of social reality. Crawford noticed several decades ago that when a person experiences a sense of loss of influence on the course of events (especially in a situation where their previous lifestyle is threatened), the need for personal control increases [ 123]. This applies especially to young people, in whom this structure is just forming, along with the development of identity and a sense of autonomy. Due to the importance of the sense of agency, it may can be an important moderator of various actions taken by young people. To sum up: the sense of agency may turn out to be an important variable moderating, for example, the expectations of the workplace (also in terms of ensuring well-being).

### Remuneration in the perspective of young people and the sense of agency. The place of well-being in the structure of professional expectations

To meet their needs, basic and higher, people today must have money at their disposal, which is earned mainly by doing work and being paid for it. Remuneration is defined as a reward or payment for work performed, including various forms of payment: salaries, bonuses, allowances, health, pension, transport benefits, etc. It is part of a broader compensation system that includes, among other things, base wages, individual bonuses, deferred payments, and team and organizational salaries. These systems are shaped by cultural norms, employee preferences, and legal regulations [124]. By the way, in a broader context, in addition to cash benefits, remuneration may also include benefits, privileges, job satisfaction, organizational affiliation, status, and other internal and external rewards that the employee considers valuable [124]. It should be noted that wellbeing solutions dedicated to employees can also be remuneration understood in this way. Therefore, in order to avoid ambiguity, it should be clearly stated that from the point of view of our considerations, we are interested in remuneration in the sense of financial compensation received by the employee in exchange for the work performed, as pay [125,126]. It includes both the basic salary and additional components such as bonuses, performance awards or others. They perform key functions in managing people in the organization – motivational, retention and information – they signal the value of specific work in the eyes of the organization and affect the perceived fairness and employee engagement [125,126].

In microeconomic models, remuneration (understood as income from work) plays a key role in shaping the consumption, savings and investment decisions of individuals. It is treated as the main factor influencing shifts in the demand curve, which emphasizes its fundamental importance in a market economy [127]. In the context of social policy, the level of remuneration also affects access to public services, education and health care, and thus the quality of life.

From an individual point of view, remuneration primarily has an income function, enables the individual to satisfy basic life needs, and shapes his basic social and social security [128,129]. It is the main source of income for most employees and is the basis of their economic security [130]. Remuneration also has a symbolic function, being an expression of recognition, status and social position of the employee in the organizational structure. In sociological terms, wages can be thought of as a form of “symbolic capital” that affects power relations, prestige, and access to resources [131]. In the context of the expectations of Generation Alpha, which shows a high sensitivity to fairness, transparency and personalization of working conditions, remuneration as “pay” can be seen not only as a source of income, but also as an expression of recognition, status and influence on one’s own career path.

The item “high earnings/good salary/satisfactory pay” opens the lists of rankings of expectations that young employees (Alpha, as well as their slightly older colleagues – representatives of Generation Z, because these age categories are often still surveyed together) have towards current or future employers [132–135]. In a 2025 survey of American students and graduates conducted by Enpulse [136], as many as 47% of young employees indicated salary as the most important criterion for choosing a workplace, ahead of aspects such as the form of employment or the possibility of professional development. Similarly, a global report by the International Labor Organization shows that young people (15–24 years old) increasingly treat financial stability as a condition for entering the labor market. The report also includes data on people looking for their first job, indicating their difficulties in finding stable employment [137]. A report by Manpower shows that 73% of young Gen Z employees are currently expecting a salary increase. Significantly, 47% of these young people say they want to change jobs in the next six months if their expectations are not met [138].

The financial expectations of young people in the Western world – especially Gen Z and Alpha – are a complex phenomenon that is driven by a range of social, psychological and economic factors. On the one hand, the specific social and economic situation on the Polish labor market is in line with global trends. The high financial expectations of young Poles may be a manifestation of the rising cost of living, urbanization, consumer pressure and comparing themselves to their peers – both in the real world and on social media. The BIK and ANG Group report indicate that almost three out of four surveyed young adults in Poland do not feel financially secure today, which translates into a strong need for stability and higher income [139]. Financial expectations are also related to the need for quick gratification and impulsive consumption, which is confirmed by financial awareness surveys of young Poles aged 15–24. Young people often make financial decisions under the influence of emotions, trends and social pressure, which can lead to debt and difficulties in managing such an unrealistic budget [140]. Self-control is crucial to making rational decisions, especially under conditions of temptation or social pressure [141]. Since young people are just forming their financial habits, low levels of self-control may result in a preference for immediate rewards (e.g., quick pay) at the expense of financial stability in the future. Market reports indicate that young Poles have significantly higher financial expectations than it results from the realities of the labor market, they often do not agree to compromises – they reject offers below their financial expectations, even if they are in line with market standards. Some pupils and students are supported by theirs families, which allows them to look for the “ideal” job for longer [142].

On the other hand, young people globally also declare that the amount of salary and benefits is not everything. According to researchers, well-being at work is one of the priorities of young people [57]. In recent years, we have also observed a clear change in the priorities of young employees in Poland. Expectations towards work increasingly go beyond traditional aspects such as remuneration or job stability, to include psychological comfort, life balance or supportive management style [ 143–145].Thus, according to surveys from 2025, over 40% of young respondents declare their readiness to resign from work if they cannot take care of their mental well-being, and work-life balance is one of the three most frequently indicated criteria when choosing their first job [146]. Alphas expect to do well in all aspect of life, are raised in material prosperity and do not tolerate obstacles that hinder the satisfaction of their needs or limit their comfort, including in the workplace [39].

The modern labor market, especially among young generations, is characterized by a simultaneous increase in expectations regarding remuneration and the quality of the work environment, which can be identified with care for wellbeing. Although at first glance this may seem contradictory – because high wages are often associated with intense work and stress – in reality, these two areas increasingly coexist as complementary elements of an attractive workplace. Referring to Maslow’s hierarchical model [147], higher-order needs (e.g., work-life balance, emotional well-being) can only be met if lower-order needs (e.g., subsistence minimum, met by wages) are met. Young workers expect work to be not only profitable, but also sustainable, supportive and meaningful. Companies that offer high wages but ignore wellbeing lose out on retention and reputation. On the other hand, organizations that combine competitive remuneration with a culture that supports well-being gain a competitive advantage (Google, Netflix or Polish technology startups that implement wellbeing strategies as an element of employer branding). Therefore, it can be assumed that young people with high salary expectations will also look for a job where wellbeing is cared for. This assumption led us to formulate the **first main hypothesis: (H1)** Expecting an employer to provide satisfactory remuneration is associated with searching for an employer who will ensure well-being.

When talking about employee well-being, we took into account the division into physical, emotional and other well-being, which is repeated in many of the concepts signaled earlier in the review. The separation of physical and emotional well-being is well rooted in theory [76,77,80,81,83]. We did not decide to isolate in our research, for example, the area of spiritual well-being [82,83], assuming that it may be less tangible for young people; Instead, we proposed the area of work-life balance, which is so important for the young generation [14,135] and comfortable working conditions [13,48]. Following this lead, we have clarified the hypotheses related to the first main hypothesis: (H1a) Expecting an employer to provide satisfactory remuneration is associated with searching for an employer who provides comfortable working conditions; (H1b) Expecting an employer to provide satisfactory remuneration is associated with searching for an employer who will ensure physical well-being; (H1c) Expecting an employer to provide satisfactory remuneration is associated with seeking an employer who ensures emotional well-being; (H1d) Expecting an employer to provide satisfactory remuneration is associated with seeking an employer who ensures work-life balance.

As we have shown earlier in the literature review, according to many researchers, self-efficacy is a mediating factor between an individual’s goals and his or her efforts to achieve them [19,148]. Therefore, we formulate the **second main hypothesis: (H2)** High expectations of satisfactory remuneration are more strongly associated with searching for an employer who will ensure well-being in people with a high sense of agency.

Similarly, when speaking about well-being expectations, we have referred to the areas indicated earlier and we have specified hypotheses related to the second main hypothesis in turn: (H2a) High expectations regarding satisfactory remuneration are more strongly associated with searching for an employer who will provide comfortable working conditions in people with a high sense of agency; (H2b) High expectations regarding satisfactory remuneration are more strongly associated with searching for an employer who will ensure physical well-being in people with a high sense of agency; (H2c) High expectations regarding satisfactory remuneration are more strongly associated with searching for an employer who will ensure emotional well-being in people with a high sense of agency; (H2d) High expectations regarding satisfactory remuneration are more strongly associated with searching for an employer who will ensure work-life balance in people with a high sense of agency.

## Methods

### Research model and operationalization of variables

The aim of the presented study was to find answers to the questions about the requirements for remuneration and well-being at work that young people have, whether these two groups of expectations are causally related, and what role the sense of agency of Generation Alpha representatives plays in this causal system. In addition, we wanted to check whether the model built from the above variables meets the requirements of reliability and validity.

The study used a quantitative research method using the author’s questionnaire. The choice of the quantitative method in this study was dictated by the need to capture general trends and preferences of the surveyed representatives of Generation Alpha, which may be important for future human resource management strategies. Quantitative methods allow for structured data collection, which ensures comparability of results and enables a statistical analysis of the relationships between variables [149]. The operationalization of variables was made on the basis of concepts and research presented in the literature review. Since the planned research process required the development of a measurement questionnaire, the selection of specific questionnaire items assigned to individual constructs was based on a selective approach.

In our model, we considered not only the expectations of young people regarding (1) basic salary (guaranteed by the employment contract), but also (2) additional financial benefits (i.e., bonuses and cash prizes), and (3) other benefits (such as additional medical care, group insurance). The components of the salary construct (S) were described by items S23, S24, S25.This is in accordance with the criteria functioning in Polish labor law or business practice [129].

In our model, we included four areas that make up the construct of well-being. And so, in the construct of well-being, physical well-being is described by items PW11 and PW12, which refer to the employer’s support in the field of physical activity and diet, in accordance with the understanding of wellness as harmony of body, mind and spirit [68,82]. Emotional well-being has been described in items EW13, EW14 and EW22, which reflect support in coping with stress, personal development and the work atmosphere – similarly to the concept of mental and social well-being [63,80] Work comfort (CW) and the items CW8 and CW57 describing it concern ergonomics and the possibility of personalizing the workspace, which is in line with the definitions of physical well-being and the sense of impact on the work environment [91].

The work-life balance (WLB) construct is quite extensive. It covers issues such as the possibility of choosing working hours (WLB5) or WLB7 additional vacation days (WLB7) relating to the flexibility of working time, which is a key component of work-life balance and a priority expectation of young people [14]. Other components of the WLB construct include the expectations of young people in terms of support in childcare (WLB9) and co-financing in this area (WLB10) and determine the need to match organizational resources to the family needs of employees [ 8,12]. Since the needs of employees are very diverse and it would be difficult to list them separately, we also referred to the general principle of the need for support in building work-life balance (WLB16), which reflects the organization’s commitment to promoting harmony between roles, in line with the view of well-being as an integrated state that allows you to cope with life circumstances [80,82,98]. Finally, we have taken into account the need to work in a place where there is acceptance of diverse lifestyles (WLB 48). This point refers to values such as inclusivity and authenticity, and is a manifestation of social and psychological well-being. An analysis of the literature shows that young people in Western culture value the opportunity to be themselves and express their own views [16,17].

Following Seligman’s perspective [82], we assumed that each of these areas could be measured independently of the others and that each contributed to well-being.Noting how important a role the sense of agency (A) plays in undertaking various actions, we introduced it as a moderating variable to our model. Item A29 links agency to the achievement of goals and tasks, which is consistent with the concept of agency as the ability to initiate actions and achieve results, and treating the effectiveness of action as a key component of the sense of agency [19,112]. Following this lead, we have also described agency as the need for influence at work (A30). Such an approach is consistent with the concept of autonomy and self-determination from [116,118]. In addition, we related agency to creativity and freedom of action (A60) [16,118]. It seems that the opportunity to be creative/pursue creative tasks at work may be a particularly expressive manifestation of agency for Generation Alpha, who are encouraged to “express themselves” from an early age.

**Fig 1:**
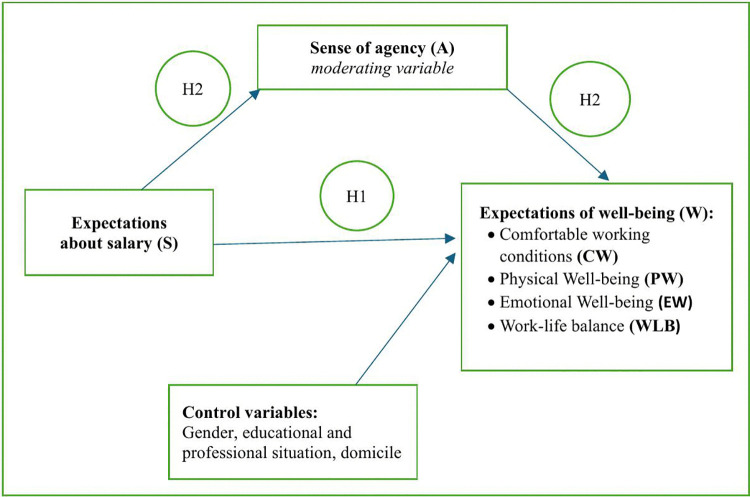
Generation Alpha’s Expectations of Remuneration and Search for Well-Being in the Workplace and Sense of agency: A Proposed Conceptual Model.

The control variables in our study were gender, housing, educational and professional situation of the respondent (Fig 1).

### Research methodology

The research was conducted in Poland in 2023 among a group of 446 representatives of Generation Alpha (221 female, 225 male). Respondents were selected on a quota basis by age, gender and province (region) based on data from the Service of the Republic of Poland (https://dane.gov.pl). The study lasted from October 20 to November 10, 2023. During this time, respondents had access to the online survey questionnaire. Both the invitation to participate in the study and the entire communication with the participants took place by e-mail. Our participants were of legal age, and by taking the survey, they also gave their informed consent to participate in the study. They could also learn about the purpose and briefly outlined subject of our research, the planned use of the data, as well as the personal and e-mail address of the project manager, who was available in case of questions or doubts. The participants were also assured of anonymity. Participants provided informed implied consent to participate in the study by proceeding after reading the introductory information included in the questionnaire. This approach is considered acceptable in anonymous studies involving minimal risk to participants. The nature of the study (a study without collecting biological material, not interfering with the psyche of the participants) and the scope of the collected data indicated that the study did not require additional consent from the university ethics committee.

The study was conducted using the CAWI (Computer-Assisted Web Interview) technique by an external company specializing in social research (the research company was selected in accordance with the procedures in force at the university where the authors work). The choice of this technique was dictated by, m.in other things, the specificity of the target group – Generation Alpha are people brought up in a digital environment, for whom the use of the Internet and mobile devices is natural. As a result, CAWI provides them with comfort and flexibility in answering, which promotes honesty in answers. In addition, CAWI ensures quick and effective collection of large amounts of data, lower costs of conducting the survey, anonymity and a greater sense of privacy of respondents, and automatic data encoding [150]. The questionnaire contained single-choice closed-ended questions. A five-point Likert scale was used for all questions (except for the metrics questions). The questionnaire included descriptive scales from “definitely not important” (or “ definitely not”) to “ definitely important” (or “definitely yes”). The answers were assigned the following values: 1 for “ definitely not important” or “ definitely not”, 2 for “rather not important” or “rather not”, 3 - “ difficult to say”, 4 - “rather important”/ “rather yes”, 5 - “ definitely important”/ “definitely yes”.

### Model Validation

Analysis of the obtained data allowed for validation of the model. First, the reliability and validity as well as the quality of fit of the model constructs were measured, and finally the hypotheses were tested.

There were no observations with missing values; no significant outliers were observed, although the data were somewhat abnormal (two items were found with kurtosis statistics exceeding 3). The pattern is left-skewed, slightly asymmetric (skewness takes negative values, oscillating between 0 and −2), but within the normal distribution [151,152]. However, because the deviations were not significant, there was no need to exclude items. Factor analysis (Table 1) confirmed a good fit of the individual items forming the constructs of variables within the model (loadings greater than 0.5).

**Table 1:** Confirmatory factor analysis: items and loadings.

Items	Comfort Work (CW)	Well-being Physical (PW)	Emotional Well-being (EW)	Work-life balance (WLB)	Remuneration (S)
CW_57	0.617				
CW_8	0.893				
PW_12		0.626			
PW_11		0.872			
EW_14			0.708		
EW_13			0.725		
EW_22			0.764		
WLB_9				0.608	
WLB_10				0.632	
WLB_7				0.692	
WLB_14				0.708	
WLB_48				0.712	
WLB_5				0.733	
S_23					0.748
S_25					0.755
S_24					0.791

The reliability and validity (convergent and discriminant) of individual constructs in this study were assessed using the following measures: standardized Cronbach ‘s alpha, composite reliability, average variance extracted (AVE), and the Fornell–Lacker criterion.

Satisfactory Cronbach’s Alpha results ensure that all items in the test measure the same concept, and the construct has internal consistency. All the constructs examined obtained values above 0.7 (for CW = 0.71; PW = 0.71; EW = 0.78) or 0.8 (for WLB = 0.84; S = 0.82). We assume that all dimensions of the construct meet the typical requirements.

However, Cronbach’s alpha, given the heterogeneity of the scales in our study, may prove to be an insufficient measure of test reliability. We therefore used a composite reliability test, which measures the internal consistency and reliability of the construct, considering the common variance between items and measurement errors. As in the previous analysis, all the tested constructs obtained satisfactory values above 0.7 (*CR* for CW = 0.74; PW = 0.72; EW = 0.77) or 0.8 (CR for WLB = 0.84; S = 0.81).

Convergent validity was estimated using the average variance extracted (AVE). For each construct, except one, the AVE is greater than 0.5. (AVE for CW is 0.59; PW = 0.57; EW = 0.534; S = 0.56) Only for WLB the AVE is 0.47, but as higher than 0.4, it is acceptable [153].

We assessed discriminant validity using the Fornell-Larcker criterion [154], checking whether items belonging to different constructs that should not be related are in fact not related. Based on this analysis, one can assume a low correlation between the results of measurements obtained using scales measuring different variables. According to the Fornell-Larcker criterion, the bold square root values of AVE are higher than their highest correlation with any other construct (Table 2).

**Table 2:** Discriminant validity of the model – Fornell-Larcker criterion.

	EW	PW	CW	S	WLB
**EW**	**0.733**				
**PW**	0.176	**0.759**			
**CW**	0.234	0.155	**0.768**		
**S**	0.516	0.341	0.454	**0.765**	
**WLB**	0.206	0.136	0.181	0.399	**0.682**

The analyses performed indicate that the measurement model is characterized by satisfactory reliability and validity, which allows us to proceed to further estimation. Confirmatory factor analysis (CFA) was used for this purpose, because, as shown above, we found grounds for formulating hypotheses on the relationships between the studied variables (model constructs). Finally, our results show that the fit index for our measurement model is an approximate fit: *χ 2* = 341.041 (*df* = 100), *p* = 0.000 < 0.005 – reject the model; *χ 2*/ *df* = 3.41 < 5 – good fit. GFI = 0.904 > 0.9 – good fit; AGFI = 0.897 ≥ 0.9 – good fit; RMSEA = 0.074 < 0.08 –fair fit; SRMR = 0.074 < 0.08 – good fit. If the chi-squared test rejects the model but *SRMR* ≤ 0.08 and all standardized residuals are small (i.e., there are no large residuals), then we can claim the model fits approximately well [155].

## Results

### Participants characteristics

As mentioned earlier, all respondents were adults born after 2000, who were no less than 19 and no more than 22 years old in the year of the study (*M*_age_ = 20.5 to *SD* = 1.117 year). The study therefore covered adult representatives of Generation Alpha, who in our opinion may have more specific expectations regarding working conditions than their younger colleagues, and often already have their first professional experience (and this was the case, 62% of our respondents were working at the time of the study). The largest percentage of respondents (80%) were people who had completed secondary school (general or technical), then high school graduates (10%), school preparing for employment (5%) and people who had completed primary school (3%) and junior high school (2%). Our respondents are residents of villages or small towns with up to 20k inhabitants (31%), medium-sized towns or larger cities (33%) and cities with 200k inhabitants (36%).

### Analytical framework

The purpose of the study was to examine whether the expectations of young people about remuneration are related to their expectations about well-being at work, and whether sense of agency moderates this relationship.

We developed and empirically validated a model including the following constructs: remuneration expectations (S) as the independent variable; expectations regarding well-being at work as the dependent variable – operationalized through four components: work comfort (CW), physical well-being (PW), emotional well-being (EW) and work–life balance (WLB); and sense of agency (A) as a moderator of these relationships (*Model Validation* section).

Structural equation modeling (SEM) with path analysis was applied. The analyzes report standardized path coefficients (β), standard errors, *t* values, *p* values, and coefficients of determination (R²) for each dependent variable examined. The moderating role of the sense of agency was tested by introducing an interaction term (A × S; sense of agency × remuneration expectations) directly into the path model and estimating its effect for each dimension of well-being at work. To visualize the moderation effects, simple slope graphs were presented, illustrating changes in the strength of the relationship between remuneration expectations and expectations regarding individual well-being components at different levels of sense of agency.

Furthermore, the effects of control variables (age, gender, place of residence, employment status or domicile) were examined within the same path-analysis framework. Statistically significant effects of the control variables are reported in a tabular format.

### Main analyses

The main point was to examine the relationship between remuneration expectations (S) and total well-being (W) and its individual components.

The analysis showed that high expectations regarding remuneration go hand in hand with searching for an employer who will ensure well-being at work and in each of its areas (Table 3). The relationships are statistically significant (at the level of p < 0.001). The coefficient of determination *R²* for the relationship between expectations regarding remuneration and expectations regarding well-being is greater than zero (*R²* CW = 0.206; *R²* PW = 0.116; *R²* EW = 0.267; *R²* WLB = 0.159). Expectations regarding remuneration (S) therefore explain the greatest variance of expectations around emotional well-being (EW), which is 27%, and then in the area of expectations regarding comfortable working conditions (CW), accounting for 21% of the variance of this variable. Next, expectations regarding remuneration (S) account for 16% of the variance of expectations regarding work-life balance (WLB) and the weakest, because only 12% of the variance of the variable of expectations that the employer will act in the area of taking care of the physical well-being of employees (PW). These relationships are also illustrated by the path analysis (Table 3), where the strength of the causal relationship between expectations regarding remuneration and expectations regarding emotional well-being is the strongest, and the weakest in the case of expectations regarding physical well-being.

**Table 3:** Model with the inclusion of the moderating variable (A) – path analysis.

	Parameter estimates	Standard errors	T values	P values	Path coefficients (standardized)
**S - > CW**	0.875	0.115	7.614	0.000	0.454
**S - > PW**	0.558	0.099	5.611	0.000	0.341
**S - > EW**	0.501	0.062	8.128	0.000	0.516
**S - > WLB**	0.574	0.086	6.639	0.000	0.399
With **the moderator, a sense of agency (A)**					
**A - > CW**	2.428	0.504	4.816	0.000	0.464
**A x S - > CW**	−0.439	0.090	4.875	0.000	−0.666
**A - > PM**	2.072	0.395	5.245	0.000	0.524
**A x S - > PW**	−0.333	0.069	4.856	0.000	−0.673
**A - > EW**	1.638	0.268	6.120	0.000	0.611
**A x S - > EW**	−0.205	0.044	4.614	0.000	−0.626
**A - > WLB**	1.044	0.346	3.015	0.003	0.457
**A x S - > WLB**	−0.144	0.062	2.312	0.021	−0.505

Another key research goal is to examine the agency sense moderation in our model.

**Fig 2:**
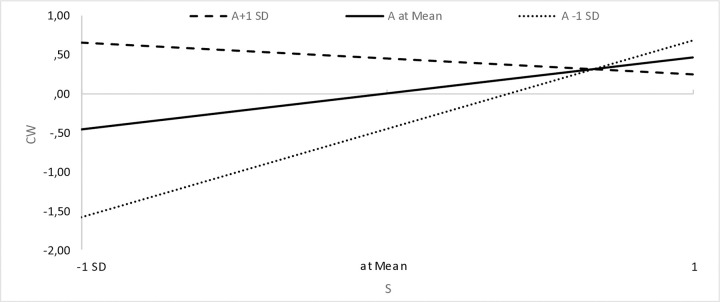
Sense of agency and the relationship between expectations regarding remuneration and comfortable working conditions.

**Fig 3:**
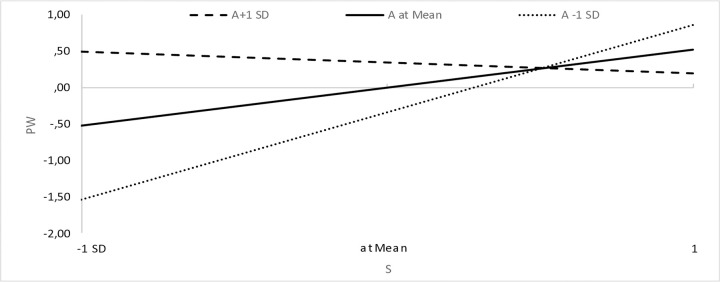
Sense of agency and the relationship between expectations regarding remuneration and physical well-being.

**Fig 4:**
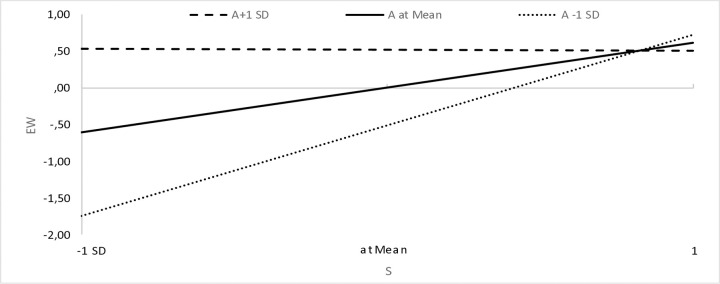
Sense of agency and the relationship between expectations regarding remuneration and emotional well-being.

**Fig 5:**
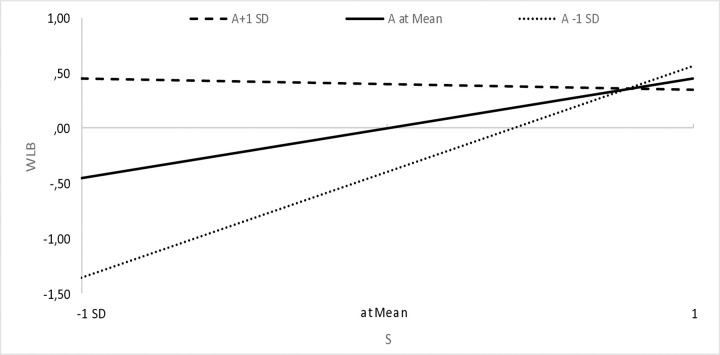
Sense of agency and the relationship between expectations regarding remuneration and work-life balance.

We assumed that a sense of agency (A) would strengthen the relationship between high pay expectations and high expectations (and interest) in an employer that cares about well-being at work. The results show that this is not the case (Table 3), but rather the opposite (the moderating variable combined with high pay expectations has a significant, negative effect on respondents’ well-being expectations). Interestingly, however, a graphical presentation of variance-based structural equation modeling plots [153] shows that when taking into account expectations of comfortable working conditions, for respondents with a high sense of agency (i.e., A + 1 standard deviation above the mean), we observe a weaker relationship (i.e., a flatter line) between comfort expectations and pay expectations than for respondents with a low sense of agency (i.e., A-1 standard deviation below the mean), the slope of which is very steep. This shows that low expectations regarding remuneration translate to a greater extent into lower expectations regarding comfortable working conditions in the case of respondents with a low sense of agency than in those with a high sense of agency (Fig 2). The same is true for the other areas of well-being, where low expectations regarding remuneration translate to a greater extent into lower expectations regarding physical well-being (Fig 3), emotional well-being (Fig 4) and work-life balance (Fig 5) in the case of respondents with a low sense of agency than in those with a high sense of agency.

Finally, we checked whether the control variables (gender, place of residence, professional work) considered by us were significantly related to the selected expectations. Table 4 presents only the significant relationships found – as can be seen, their strength is weak, but they indicate that respondents who lived with their parents at the time of the survey had lower expectations regarding comfort in the workplace or emotional well-being. The residents of large cities responded in a completely oppositely formulating such expectations more often than residents of villages and small towns.

**Table 4:** Influence of control variables (significant only).

Control variables	Parameter estimates	Standard errors	T values	P values	Path coefficients (standardized)
Living with parents - > expectations regarding comfort in the workplace	−0.316	0.111	2.843	0.005	−0.058
Living with parents - > expectations regarding emotional well-being at work	−0.117	0.057	2.064	0.040	−0.112
City - > expectations regarding comfort at work	0.088	0.034	2.587	0.010	0.052
City - > expectations regarding emotional well-being at work	0.040	0.017	2.308	0.021	0.122

## Discussion

### Expectations regarding pay and well-being in the workplace

Our research confirmed reports that remuneration is important for Generation Alpha, which was of course not surprising; such expectations are also formulated by other professionally active generations, including Generation Z, which preceded our respondents. Research conducted by the National Research Institute among several thousand students in the last grades of secondary schools, i.e., people on the threshold of adulthood, indicates that the basic desire related to work for most young Poles (71%) is high earnings [156]. The desire to earn more money is also the most common reason for looking for a new employer in the group of respondents aged 18–24 [157]. We specified that our respondents care primarily (Table 5) about a satisfactory basic salary (*M* = 4.74), which, with a very low standard deviation and maximum values of the mode and median, indicates exceptional agreement of respondents in this respect. This need for financial security seems understandable in a country hit by financial crises, the recent Covid 19 pandemic and the ongoing full-scale war in neighboring Ukraine.

**Table 5:** Model variables – descriptive results.

Variables		Items – description	Mean	Standard deviation	Dominant	Median
Well-being expectations	Work comfort (CW)	(CW8) The company offers amenities such as a desk for standing work, an ergonomic seat (e.g., a ball or a bean bag)	3.61	1.032	4	4
		(CW57) Working in a company where you can design your own workspace (choose your desk, wall graphics, color of your computer and accessories, bring flowers)	3.56	1.132	4	4
	*Physical Well-being (PW)*	(PW11) Employer support for employees’ physical activity (which, according to medical recommendations, does not harm health but has a positive effect on it);	4.29	0.747	5	4
		(PW12) Employers’ support for a balanced diet (in line with medical recommendations)	4.62	0.594	5	5
	*Emotional Well-being (EW)*	(EW13) Supporting employees in coping with stress and various types of crisis situations (related to what happens at work and outside of work)	3.09	1.063	3	3
		(EW14) Supporting employees in their personal development, not only in the professional area, but also in the development related to the passions, dreams, desires and interests of employees	4.53	0.620	5	5
		(EW22) The company’s care for good interpersonal relations/good atmosphere/good climate at work	4.61	0.628	5	5
	*WLB*	(WLB5) Possibility to choose working hours (full-time or part-time work)	4.35	0.776	5	4
		(WLB7) Employer offering additional days of paid vacation	4.15	0.798	4	4
		(WLB9) Running and co-financing by the employer of a nursery/kindergarten for employees’ children	3.18	1.082	4	3
		(WLB10) Co-financing by the employer of other forms of care for children at nursery, preschool or early school age (e.g., co-financing for the work of a nanny)	3.48	1.127	4	4
		(WLB16) Supporting employees in building work-life balance	4.23	0.914	5	4
		(WLB48) Working in a company that accepts an unconventional approach to the lives of employees (where everyone can be themselves regardless of their lifestyle or way of being)	4.27	0.880	5	4
Salary expectations *(s)*		(S23) Satisfactory basic salary	4.74	0.521	5	5
		(S24) Satisfactory additional financial components of remuneration (bonus, award, etc.)	4.55	0.661	5	5
		(S25) Satisfactory non-wage benefits (e.g., sports card, medical package, medical insurance, group insurance, etc.)	4.15	0.866	4	4
A sense of agency (A)		(A29) Performing satisfying professional tasks	4.38	0.718	5	4
		(A30) To have the feeling that my opinion is taken into account in the company	4.43	0.702	5	5
		(A60) Have a job where you can be creative	4.15	0.857	4	4

Our research also confirmed that broadly understood well-being, work comfort, and working in a friendly environment (in the emotional sense) are important for Generation Alpha. The respondents mainly expected support around a balanced diet (in line with medical recommendations) (*M* = 4.74), employer’s care for good interpersonal relations/good atmosphere and climate at work (*M* = 4.61) and flexible working hours (*M* = 4.35). The weakest expectations were those related to stress prevention (*M* = 3.09) and WLB solutions (running and co-financing a nursery/kindergarten for employees’ children by the employer – *M* = 3.18). At the same time, these items were characterized by some of the highest standard deviations, which indicates the heterogeneity of the responses, which are widely scattered from the mean – in the group of respondents there were therefore many people who definitely had such expectations (perhaps respondents planning to start a family soon or experiencing the negative effects of stress) and those for whom they were definitely unimportant. The need for flexibility and comfort at work among our respondents are quite similar to the findings of other researchers [58,59].

What we managed to confirm in the research, and which is rarely the subject of analysis, is a significant, although moderate in terms of strength, causal relationship between high expectations regarding remuneration and the formulation of expectations regarding ensuring well-being at work (Table 3). High expectations regarding remuneration explain the most variance in expectations regarding emotional well-being (*R² =* 0.267) and are least related to expectations regarding physical well-being (*R² =* 0.116). Considering the nature of the questions, respondents may have believed that such activities (e.g., elements of a healthy lifestyle) are easier for employees to plan and implement on their own, while the issues of atmosphere and emotional safety at work are more complex and require the employer’s support.

Our findings therefore allow us to accept the first hypothesis and its accompanying detailed hypotheses. They assume that young people’s expectation of satisfactory remuneration is related to the expectations (and implicitly the search for) of an employer who will ensure well-being at work (H1) – understood as comfortable working conditions (H1a), physical well-being (H1b), emotional well-being (H1c) and work-life balance (H1d)

### The role of the sense of agency in the research model

The topic of independent planning of activities is related to another variable included in our model, which is the sense of agency. We assumed that he would be the moderator of the relationship between salary expectations and well-being at work.

The topic of independent action planning is related to another variable included in our model, which is the sense of agency. We assumed that it would be a moderator of the relationship between expectations regarding remuneration and well-being at work. There are studies confirming that the sense of self-efficacy/agency affects well-being in various groups of employees [107]. As we described earlier, in our research, the sense of agency is associated with high expectations regarding emotional well-being (β = 0.611) and physical well-being (β = 0.524) and slightly weaker with the other two areas of well-being (Table 3). However, in combination with financial expectations, our assumption expressed in the second hypothesis (and its accompanying detailed hypotheses H2 ad) was not directly confirmed. A high sense of agency is even a negative moderator, weakening the relationship between high expectations regarding remuneration and well-being at work. This was a big surprise to us.

Looking for an explanation for this surprising result, one can start from the assumption that people with a high sense of agency perceive themselves as the key authors of their own actions, and therefore also the main agents of their own well-being. Therefore, they may not expect their employer to provide them with well-being, because they will take care of their own needs – regardless of external conditions [158,159].In this view, high agency may weaken the relationship between salary expectations and expectations towards the offer of well-being. Another clue may be a cultural explanation: young Poles function in a culture that combines individualistic aspirations with a high distrust of public institutions and entrepreneurs. Therefore high agency may lead to lower expectations towards the employer, because these people prefer to rely on themselves rather than on the system, which they perceive as unreliable, and employers’ declarations only as a manifestation of PR [160].

When formulating the research assumptions, we focused on a high sense of agency, while an analysis of the statements of people with a low sense of agency turned out to be a certain explanation. Namely, low expectations regarding remuneration translate to a greater extent into lower expectations regarding comfortable working conditions, physical and emotional well-being and work-life balance in the case of respondents with a low sense of agency (Figs 2-5). This certainly requires further research and is a direction worth explaining.

As for the second hypothesis (together with the detailed hypotheses), it cannot be confirmed directly, in the form in which it was formulated. However, we found premises that make it probable that there is a relationship between expectations in terms of remuneration and well-being at work and a sense of agency (H2), but they concern respondents with a low intensity of the latter variable.

## Limitations and directions further research

Although the presented study makes a theoretical and practical contribution, mainly related to the exploration of the “professional” expectations of the youngest group of players on the labor market, for which such studies are lacking, it also has several limitations that should be resolved soon. In this article, we present only part of the results of the conducted research, which touched on the broader subject of Generation Alpha’s expectations towards the employer (including issues related to authentic leadership, the organization’s activities in accordance with the idea of corporate sustainability or supporting employees with specific difficulties).. We hope that our research will contribute to creating a picture of young Poles’ expectations towards their workplace and employer.

We are also aware that labeling generations using dates of birth raises many doubts and it is impossible to establish an absolute boundary that would separate one generational group from the next (as is the case with Generation Alpha), and this issue may be additionally complicated by the cultural, economic and geopolitical specificity in various countries. Our findings were developed and are analyzed in the Polish context, and we recognize that next-generation workers may have a different approach to their careers than their peers in other countries.

The results of our study, due to the quota sampling (classified as non-random sampling methods), can be generalized to the entire population of young Poles (aged 19–22) only to a limited extent. The group we studied is difficult to access as part of random selection due to organizational, ethical and formal constraints (e.g., the need to obtain institutional approvals, difficulties in reaching students in different types of schools). Therefore, the quota selection we used is a kind of compromise – although it made it possible to take into account specific demographic characteristics of the respondents (such as age, gender, type of school, place of residence), while maintaining control over the sample structure, it obviously limits the scope of inference possibilities. In addition, the possibility of generalizations is complicated by the context of the environment from which the study participants originate. The level of development of the labor market in Poland, the structure of remuneration, the degree of dissemination of corporate programs supporting well-being or dominant cultural values (e.g., individualism, high value of professional work and work-life balance, low level of trust in institutions and authorities) could have influenced the way respondents understand the questions and how they value individual aspects of work. For example, in Poland, there is a paradox of inflated educational aspirations on the part of parents of today’s Alpha generation (and young people themselves), and the educational system still operates in the post-transformation model, with a strong emphasis on general education, which is not in line with the needs of the labor market [161]. This can lead to frustration for an entire generation and a reduced sense of agency when the professional reality does not meet expectations.

The limitations of our study also concern the tool itself, in which we used closed-ended questions, which involve suggesting answers to the respondent. This can be particularly dangerous when they do not have a sufficiently developed opinion on the issue raised in the questionnaire, and this could be the case with very young people, some of whom (as we assume) may not yet have specific opinions on their desired place of work. Another limitation was that as researchers we did not have full control over the implementation of the study, because the service of obtaining survey data was provided by an external entity.

Of course, our research does not exhaust the subject of Gen A expectations toward work and employers. It is only a contribution to the discussion on of the values, needs, or expectations of the youngest participants in the labor market. Generalization of the results beyond the Polish context should be made carefully and taking into account cultural and methodological differences. However, we hope that despite the imperfections of the quota selection of participants, we have gained some insight into the expectations of young people. The study can be a starting point for international comparisons, especially if it is replicated in other countries, using the tool after its cultural adaptation.

## Conclusions

Our research can serve as a starting point for research by scientists who can expand on the additional values expected by Generation Alpha. We explore the expectations of Generation Alpha representatives, so far analyzed mainly in the context of students, children, or recipients of products aimed at youth [9,47,162].

We have established that the expectations of the youngest players on the labor market go in many directions – firstly, towards earning really “good” money (satisfying basic salary plus salary supplements, bonuses), secondly – emotional and physical comfort at work is important for young people. Representatives of Generation Alpha expect their employers to take care of their well-being in the workplace, and such expectations are mainly formulated by people with a high sense of agency. It seems that there will be many such people in Generation Alpha – researchers note that the youngest employees prefer to work freely and independently. When assessing work, they not only pay attention to remuneration, but also consider whether they can have a say in the work in order to fully use their talents Generation Alpha, convinced of their own agency, and strongly focused on development [57] and benefits at work, requires a reorientation of people management strategies in organizations. For younger generations, the value of work itself is decreasing (with particular emphasis on intrinsic work values), e.g., Generation X has a stronger work ethic compared to Millennials, and Millennials – compared to Generation Z [163] which means that traditional recruitment strategies and later on, adaptation or motivation strategies may fail in the case of the youngest employees. Our research can therefore be used by employers for whom Generation Alpha is the target group of candidates for employment. The research results related to the expectations of this generation are also a source of knowledge for organizations that are currently (or will be in the near future) workplaces for Alpha. Organizations are already employing well-being managers, which seems to be a good direction [57].

## Supporting information

S1 Appendix Supporting Information Dataset – the file contains anonymized data used for analysis. (XLSX)
